# A randomized clinical trial to evaluate the stain removal efficacy of a sodium phytate dentifrice formulation

**DOI:** 10.1111/jerd.12355

**Published:** 2018-02-07

**Authors:** Kimberly R. Milleman, Jonathan E. Creeth, Gary R. Burnett, Jeffery L. Milleman

**Affiliations:** ^1^ Salus Research Fort Wayne Indiana 46825; ^2^ GSK Consumer Healthcare Weybridge Surrey, KT13 ODE United Kingdom

**Keywords:** extrinsic dental stain, relative dentin abrasivity, sodium phytate toothpaste

## Abstract

**Objectives:**

Phytate is an organic, cyclic polyphosphate analogous to linear condensed polyphosphates used as stain removal agents. This study investigated stain removal efficacy of an experimental sodium phytate‐containing dentifrice compared to a reference dentifrice.

**Methods:**

An experimental, moderate abrasivity (relative dentine abrasivity [RDA] ∼130) antisensitivity fluoride dentifrice containing sodium phytate (0.85% w/w as the hexasodium salt) (*n* = 111) was compared to a reference, marketed, low‐abrasivity (RDA ∼ 43), anti‐sensitivity fluoride dentifrice (*n* = 113), both containing 1150 ppm fluoride as sodium fluoride. Primary efficacy variables were between‐treatment differences in extrinsic dental stain of anterior teeth after 6 and 12 weeks' twice‐daily use, using Lobene stain index (MacPherson modification, MLSI) mean area (*A*) and intensity (*I*) scores. Comparisons included whole‐tooth and hard‐to‐reach areas (gingival, interproximal, body of lingual).

**Results:**

At both 6‐ and 12‐week timepoints, MLSI (*A* × *I*) scores for total area and hard‐to‐reach areas for the experimental dentifrice were statistically significantly lower than baseline (*P* < .0001 for all). This was demonstrated for the reference dentifrice at 6 weeks only, for total, interproximal (*P* < .0001 for both), and body of lingual (*P* = .0395) scores. Compared with the reference, the experimental dentifrice had statistically significantly lower MLSI scores at both 6 and 12 weeks for all outcome variables including both total MLSI (*A* × *I*) and hard‐to reach areas (*P* < .0001 in all cases). Products were generally well‐tolerated.

**Conclusions:**

Differences between treatments were considered clinically differentiable. Sodium phytate may therefore be a suitable additive ingredient to improve tooth stain control performance within an otherwise conventional dentifrice formulation.

**Clinical Significance:**

Following 6 and 12 weeks brushing, clinically differentiable differences were shown in stain index scores with an experimental dentifrice containing sodium phytate compared to a reference dentifrice without sodium phytate. Sodium phytate may therefore be a suitable additive ingredient to improve tooth stain control performance within an otherwise conventional dentifrice formulation.

## 1 INTRODUCTION

An important function of a dentifrice formulation is to control extrinsic dental stain formation caused by certain dietary components, tobacco or medications that bind to proteinaceous compounds found in the salivary enamel pellicle.^1^, ^2^, ^3^ Currently, in almost all dentifrices this function is delivered primarily by incorporating dental‐grade particulate abrasives. These use a physical mode of action to remove stains during tooth brushing and thereby help prevent stain build‐up.^4^, ^5^ Stain removal from tooth surfaces by toothbrushing can be augmented by various chemical approaches: most commonly, dentifrices incorporate surfactants to help dislodge and solubilize stain.^5^ Research has determined that electrostatic interactions associated with protein adhesion to enamel and hydroxyapatite are important to stain deposition.^6^, ^7^ Therefore, soluble ionic agents that interfere with protein binding to tooth surfaces are also routinely incorporated in dentifrice formulations to provide additional extrinsic dental stain control benefits. The most commonly used examples are linear condensed polyphosphates.^8^, ^9^ Such soluble agents have two principle advantages: (i) they should function without substantially increasing abrasivity, as would result from achieving the same increase in stain removal by simply boosting the amount of abrasive used,^9^ and (ii) they should also increase cleaning in inaccessible regions of the dentition where toothbrush bristles may fail to reach. This lower‐abrasivity approach to achieve effective extrinsic stain removal may be particularly important for people at risk from hypersensitivity due to dentin exposure from erosive toothwear, as higher abrasive dentifrices could potentially exacerbate the condition.^10^, ^11^

Pyrophosphate, tripolyphosphate, and hexametaphosphate are examples of linear, condensed polyphosphates that are now widely used in dentifrices. They have a strong affinity for calcium ions due to their chelating ability, and hence have a strong affinity for the hydroxyapatite surface of enamel.^9^ Polyphosphates were first introduced into dentifrices to reduce development of dental calculus, as they are believed to block calcium sites for crystal growth by adsorbing onto the surfaces of hydroxyapatite crystals.^12^, ^13^ This mechanism of action has also been utilized in the development of anticalculus chewing gum.^14^ The ability of polyphosphates to bind to tooth surfaces has also been shown to inhibit protein adsorption,^12^, ^13^ most likely by reducing the adhesion force of adsorbed pellicle proteins.^15^ Their ability to chelate calcium may reduce ionic crosslinking of pellicle and stain molecules, inhibiting stain build‐up once a pellicle is formed.

Sodium phytate is a representative of a different class of polyphosphates: it is a cyclic, organic hexa‐phosphate and is the principal phosphorus storage molecule in seeds and grains.^16^ It therefore shares some chemical commonality with the inorganic linear condensed polyphosphates typically used in oral care products, by virtue of the presence of multiple phosphate groups. A clinical study found that use of a phytate‐containing mouthwash reduced dental calculus formation.^17^ However, there appear to be no literature reports of the ability of dentifrice formulations containing phytate, in addition to a conventional abrasive system, to reduce extrinsic tooth stain.

The aim of this study was therefore to investigate the stain control potential over 12 weeks of twice‐daily use of an experimental dentifrice containing phytate in a conventional moderate‐abrasivity silica base. The stain removal efficacy of this dentifrice formulation incorporating sodium phytate (0.85% w/w as the hexa‐sodium salt) was compared with that of a marketed, low abrasivity daily use dentifrice without specific ingredients to enhance stain removal, as a reference. Both the reference control and the experimental dentifrice contained 1150 ppm fluoride as sodium fluoride (NaF) and 5% w/w KNO_3_ to provide relief from dentin hypersensitivity. Oral tolerability was also evaluated over the course of the study.

## 2 METHODS

This was a 12‐week, single‐center, randomized, single examiner‐blind, stratified, two‐treatment, parallel‐group study in healthy volunteers. The study was conducted at Salus Research, Fort Wayne, IN. The study protocol was approved by an independent institutional review board (Number: U.S. IRB2015SRI/10; U.S. Institutional Review Board, Miami, FL, USA) and the study procedures were performed in accordance with Good Clinical Practice, the Declaration of Helsinki and relevant local laws and regulations. There was one minor, administrative amendment to the protocol that did not affect study flow or outcomes.

### 2.1 Participants

The study population was recruited by the contract research organization utilizing their categorized local volunteer database. Participants were at least 18 years of age and in good general health without any significant or relevant medical or oral/dental abnormalities, with at least 16 permanent natural teeth, including 11 of the 12 anterior teeth (incisors and canines) with all lingual mandibular surfaces intact and with presence of extrinsic stain on the facial surfaces of the anterior teeth (ie, total Macpherson modification of the Lobene stain index [MLSI] area x intensity [*A* × *I*] score of ≥ 15.

General exclusion criteria included: pregnancy; breast feeding; current/relevant history of a medical condition or concomitant medication that could confound study results or pose unwarranted risk in study product administration; hypersensitivity/intolerance to study materials; use of minocycline, tetracycline or doxycycline within 30 days of screening or between screening and baseline; use of any investigational drugs or oral care products or participation in another clinical trial within 30 days of screening.

Oral exclusion criteria included: current active caries, severe gingivitis or advanced periodontal disease; dental prophylaxis within 8 weeks of screening; a nonvital anterior tooth; orthodontic bands or cracked enamel; teeth with surface irregularities, tetracycline stain, restorations or hypo/hyperplastic areas that could impact stain grading; and use of any professionally dispensed or over the counter whitening products (excluding dentifrice) within the past 3 months.

### 2.2 Clinical procedures and study products

At the screening visit, participants provided written informed consent to participate in the study. Demography, medical history and concomitant medications were recorded, followed by an oral soft tissue (OST) examination, an oral hard tissue examination and a gross visual assessment of extrinsic dental stain to confirm sufficient levels of dental stain. Participants meeting all other study criteria were considered eligible and continued to use their own dentifrice at home until the baseline visit.

At the baseline visit, participants had an OST examination followed by a 30 s brushing of their anterior teeth with water. Extrinsic dental stain assessments were carried out on the facial surfaces of the six maxillary and six mandibular anterior teeth and the lingual surfaces of the six mandibular anterior teeth, using the MLSI^18^ to score both area (*A*) and intensity (*I*) of stain (see below). Participants with a total MLSI (*A* × *I*) score of ≥15 for the facial surfaces of the 12 anterior teeth, who continued to meet all study criteria, were stratified according to their MLSI score (low < 45; high ≥ 45) and smoking status (smoker/nonsmoker) and randomized to treatment. A unique screening number identified each screened subjects, assigned in ascending numerical order as subjects signed the consent form. Subjects who met all inclusion and exclusion criteria were randomized by study site staff according to a randomization schedule provided by the Biostatistics Department of GSK Consumer Healthcare. Randomization numbers were assigned by study site staff in ascending numerical order as each participant was determined to be fully eligible and according to MLSI score strata (<45, ≥45) and smoking status (yes, no).

Participants abstained from teeth brushing or use of any oral hygiene product for at least 6 h and from eating or drinking for at least 2 h (with the exception of water) prior to each study visit until the assessments were complete. Participants were not allowed to use mouthrinse, receive a dental prophylaxis, or use any professional or over the counter whiteners during the course of the study. They were also informed that daily flossing was prohibited on the anterior teeth except for the removal of impacted food. Flossing posterior teeth was permitted.

Participants were randomized to either an experimental dentifrice containing 0.85% w/w hexa‐sodium phytate, 5% w/w KNO_3_ and 1150 ppm fluoride as NaF, with relative dentine abrasivity (RDA) measured at approximately 130 (GSK Consumer Healthcare, data on file), or a reference dentifrice containing 5% w/w KNO_3_ and 1150 ppm fluoride as NaF, with RDA measured at approximately 43 (GSK Consumer Healthcare, data on file) (Sensodyne™ Pronamel^®^, GSK Consumer Healthcare, Weybridge, UK; US‐marketed dentifrice). The examiner, study statistician, data management staff, and other employees of the sponsor who may have influenced study outcomes were blinded to treatment allocation. The blind was maintained by over‐wrapping the test product tubes in white vinyl.

Study personnel demonstrated the amount of toothpaste to be used (approximately 1.5 g), familiarized participants with the timer, and supervised the first brushing. Thereafter, participants brushed their teeth at home twice daily for one timed minute in their usual manner with their assigned toothpaste and a supplied toothbrush (Aquafresh^®^ Clean Control [Everyday Clean] Toothbrush; GSK Consumer Healthcare; UK marketed product). Participants received verbal and written product usage instructions and were asked to record each product use in a home use diary and to note any significant changes to diet or smoking status during the course of the study.

Participants returned to the clinic after 6 and 12 weeks of treatment for dental stain assessment. Replicate examinations were performed by the clinical examiner at each assessment visit with 10–30 min between repeatability assessments with a preference to score the next participant before the replicate exam. One participant per session (morning/afternoon) was chosen for repeatability assessments by the recorder at random from the participants in attendance.

Adverse events (AEs) and any abnormalities in the OST examination were recorded from the time of supervised brushing at the screening visit until 5 days after the last administration of study product. Clinical judgment was used to assess the relationship to the study product of any AE.

### 2.3 Scoring procedures: MLSI

To facilitate assessment standardization, stain assessments were performed by a single clinical examiner, in the same room, with consistent light levels throughout the study. To remove any external debris, prior to each stain assessment participants brushed their anterior teeth with a wetted toothbrush for 30 s. Teeth were air dried prior to the assessment and during the assessment as needed.

The facial and lingual surfaces of each assessable tooth were divided into four regions: *Gingival *= a crescent‐shaped band, approximately 2 mm wide, adjacent to the free margin of the gingiva and extending to the crest of the interdental papillae of the adjacent teeth; *Body *= the rest of the tooth, subdivided into distal, body, and mesial areas on the facial and lingual surfaces. For analysis, the interproximal area included only the distal and mesial body areas. Extrinsic dental stain was scored in each tooth area using the MSLI, with grades of 0–3 assigned for each category of area (*A*) and intensity (*I*), scored separately for each area of each assessable tooth: *intensity*: 0 = no stain; 1 = light stain; 2 = moderate stain; 3 = heavy stain; *area*: 0 = no stain; 1 = stain covering up to one third of region; 2 = stain covering up to two thirds of region; 3 = stain covering more than two thirds of region. The MLSI (*A* × *I*) score for each tooth area was calculated for each participant by averaging the score of the facial surfaces of the six maxillary and six mandibular anterior teeth, and the lingual surfaces of the six mandibular anterior teeth. ‘Hard‐to‐reach’ areas (based on Vorwerk and colleagues^19^) were also assessed: gingival, interproximal, body of lingual.

### 2.4 Statistical analysis

A sufficient number of participants were screened so that a maximum of 250 (approximately 125 per treatment group) could be randomized to treatment, ensuring that approximately 100 evaluable participants per group completed the week 12 assessment (based on Young and colleagues^20^). With 100 participants per group completing, the study would have 80% overall power to detect a difference for the change from baseline in overall MLSI (*A* × *I*) after 12 weeks of brushing for the comparison of the test and reference dentifrices with 80% power. This was based on the two‐sided, two‐sample *t* test at the 5% level with the estimate of variance as .04 A difference of .08 units was considered appropriate as a threshold difference between treatments for chemical cleaning‐based stain removal and prevention technologies (GSK Consumer Healthcare, data on file).

The safety analysis was performed in the safety population, defined as all randomized participants who received at least one dose of the study treatment. The efficacy analysis was performed on the intent‐to‐treat (ITT) population, defined as all participants who received the study treatment and had at least one postbaseline efficacy measurement. The per‐protocol (PP) population was defined as those participants in the ITT population for whom all postbaseline primary efficacy data were not deemed to be affected by protocol violations. The PP analysis was only scheduled to be performed for the primary endpoint if more than 10% of the participants in the ITT population were removed from the PP population.

The primary objective of this study was to compare the change from baseline in dental stain reduction between an experimental sodium phytate dentifrice and a reference control dentifrice following 12 weeks of regular brushing, as indicated by MLSI scores. Secondary objectives were to compare as above the dentifrices at 6 weeks, to evaluate and compare the MLSI scores for gingival, interproximal and body surfaces at 6 and 12 weeks, and to determine the oral tolerance of the dentifrices. All outcome variables were analyzed under a null hypothesis of no difference between study treatments against an alternative hypothesis of a difference between study treatments.

An analysis of covariance (ANCOVA) model was used to analyze the variables at each time point. The model terms included were treatment and smoking status as factors, and the participant's mean preprophylaxis MLSI score as a covariate. The MLSI stratification factor was not included as the actual baseline values were included in the model. The assumptions underlying the ANCOVA models were examined. There were no gross deviations from normality or equal variance assumptions.

A weighted Kappa coefficient was calculated to assess the intra‐examiner reliability in terms of MLSI scoring. Reliability was considered to be excellent if Kappa was >0.75, fair to good if Kappa was ≤0.75 to ≥0.4 and poor if Kappa was <0.4.

## 3 RESULTS

**Figure 1:**
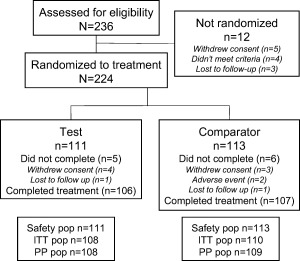
Study flow

The first participant was recruited into the study on August 24, 2015 with the last participant completing on December 1, 2015. A total of 236 participants were screened and 224 were randomized to treatment and included in the safety population (Figure 1). Baseline demographics and characteristics of the safety population are summarized in Table 1. The study population consisted of 162 (72.3%) female and 62 (27.7%) male participants with a mean age of 48.0 years (standard deviation 13.67; range 19–80) who were predominantly white (86.6%). The demographic characteristics were similarly distributed for both treatment groups. Demographic characteristics for the ITT population were similar to the safety population. PP analysis was not performed as less than 10% of participants were excluded.

**Table 1:** Baseline demographics and characteristics (safety population)

	Test (*n* = 111)	Reference (*n* = 113)
Gender, *n* (%)		
Female	73 (65.8)	89 (78.8)
Male	38 (34.2)	24 (21.2)
Race, *n* (%)		
White	96 (86.5)	98 (86.7)
Black/African‐American	10 (9.0)	12 (10.6)
Other	5 (4.5)	3 (2.7)
Mean age		
Years (SD)	47.0 (13.71)	49.0 (13.63)
Range	21–80	19–77
Total MLSI (*A* × *I*)		
<45	92 (82.9)	92 (81.4)
≥45	19 (17.1)	21 (18.6)
Smoker, *n* (%)		
No	93 (83.8)	93 (82.3)
Yes	18 (16.2)	20 (17.7)

**Figure 2:**
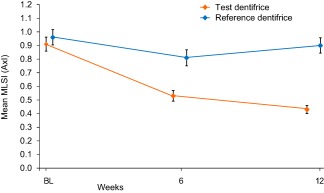
Total overall MLSI raw mean scores (±SE) (*A* × *I*) (ITT population). Data are offset for clarity

Figure 2 shows the raw mean total MLSI (*A* × *I*) scores with Table 2 showing the adjusted mean differences from baseline and differences between treatments at each time point. The test dentifrice group demonstrated statistically significant reductions in stain at both the 6‐ and 12‐week time points compared to baseline levels (*P *< .0001 in all cases). All differences were greater than the 0.08 units considered clinically differentiable. The MLSI (*A* × *I*) scores for the reference dentifrice group initially dropped, with a statistically significant change from baseline at 6 weeks (*P* < .0001), but scores at 12 weeks were no longer significantly different from baseline. Comparing the products at both 6 and 12 weeks, there was a statistically significant difference in MLSI (*A* × *I*) scores between the test and reference dentifrices, favoring the test (*P* < .0001 for both time points) with differences greater than the 0.08 units considered clinically differentiable.

**Table 2:** MLSI (*A *× *I*) adjusted mean change from baseline and difference scores at each timepoint (ITT population)

		Adjusted mean change from baseline (95% CI) *P* value		Test versus reference
MLSI	Week	Test (*n* = 108)	Reference (*n* = 110)	Difference^a^ (95% CI) *P* value
Total	6	−0.38 (–0.43, −0.33) **<.0001**	−0.14 (–0.19, −0.09) **<.0001**	−0.24 (–0.31, −0.17) **<.0001**
	12	−0.47 (–0.52, −0.41) **<.0001**	−0.03 (–0.08, 0.03) 0.3522	−0.44 (–0.52, −0.36) **<.0001**
Gingival	6	−0.16 (–0.21, −0.12) **<.0001**	−0.03 (–0.07, 0.02) 0.2903	−0.14 (–0.20, −0.07) **<.0001**
	12	−0.18 (–0.23, −0.14) **<.0001**	0.02 (–0.02, 0.07) 0.3194	−0.21 (–0.27, −0.14)** <.0001**
Interproximal	6	−0.61 (–0.69, −0.54) **<.0001**	−0.26 (–0.33, −0.18) **<.0001**	−0.36 (–0.46, −0.25) **<.0001**
	12	−0.77 (–0.85, −0.69) **<.0001**	−0.08 (–0.15, 0.00) 0.0514	−0.69 (–0.80, −0.58) **<.0001**
Body of lingual	6	−0.36 (–0.44, −0.27) **<.0001**	−0.09 (–0.17, −0.00) **0.0395**	−0.27 (–0.39, −0.15) **<.0001**
	12	−0.39 (–0.49, −0.30) **<.0001**	0.03 (–0.06, 0.13) 0.4797	−0.43 (–0.56, −0.29) **<.0001**
Body of facial	6	−0.02 (–0.04, −0.00) **0.0275**	0.01 (–0.00, 0.03) 0.1602	−0.03 (–0.06, −0.01) **0.0111**
	12	−0.02 (–0.04, −0.00) 0.0624	0.02 (0.00, 0.04) **0.0211**	−0.04 (–0.07, −0.01) **0.0034**

*P* values in bold are statistically significant (*P* < 0.05).

a From ANCOVA analysis; a negative difference favors test dentifrice.

**Figure 3:**
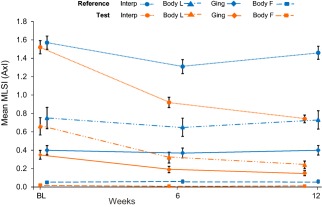
MLSI raw mean scores (±SE) (*A* × *I*) of hard‐to‐reach areas and body of facial (ITT population). Data are offset for clarity. Interp: interproximal; Body L: body of lingual; Ging: gingival; Body F: body of facial

Figure 3 shows the raw mean MLSI (*A* × *I*) scores for the hard‐to‐reach and body of facial areas with Table 2 showing the adjusted mean differences from baseline and differences between treatments at each time point. For the test dentifrice at both time points, all hard‐to‐reach areas (gingival, interproximal, body of lingual) were statistically significantly below baseline values (*P* < .0001) with the largest differences shown in the interproximal area. For the reference dentifrice, although stain levels in interproximal (*P* < .0001) and body of lingual (*P* = .0395) areas were reduced from baseline after 6 weeks, none of the other hard‐to‐reach area stain values was significantly different from baseline or had values greater than the 0.08 score deemed clinically differentiable. There were no statistically significant differences from baseline after 12 weeks' use. Comparing the products, there were statistically significant differences between the test and reference dentifrices (*P* < .0001), favoring the former, for all hard‐to‐reach areas at both time‐points. All these differences were considered clinically differentiable.

The repeatability analyses of the MLSI showed excellent agreement between the repeated assessments with kappa values for Area and Intensity of κ = 0.94 (95% CI 0.93, 0.95) for both.

Treatment‐emergent AEs (TEAEs) are summarized in Table 3. Overall, a total of nine TEAEs were reported by nine (4%) participants. Of these, four were oral and five were nonoral. None of the TEAEs reported by participants in the test dentifrice group was considered treatment‐related. Four (1.8%) participants in the reference dentifrice group reported four treatment‐related TEAEs: sensitivity of teeth (moderate), dry mouth (mild), tooth discoloration (mild), and lip ulceration (moderate). Two of the oral TEAEs (sensitivity of teeth and tooth discoloration) led to participant withdrawal. There were no serious adverse events.

**Table 3:** Treatment‐emergent adverse events (TEAE) (safety population)

	Test (*n* = 111)		Reference (*n* = 113)	
	*n* (%)	nAE	*n* (%)	nAE
At least one TEAE	2 (1.8)	2	7 (6.2)	7
Oral TEAE	0	0	4 (3.5)	4
Treatment‐related TEAE				
Dry mouth	0	0	1 (0.9)	1
Lip ulceration	0	0	1 (0.9)	1
Sensitivity of teeth	0	0	1 (0.9)	1
Tooth discoloration	0	0	1 (0.9)	1

*n* (%) = number (percent) of participants; nAE = number of TEAEs.

## 4 DISCUSSION

This study has demonstrated that a moderate abrasivity dentifrice formulation containing phytate as a soluble stain control agent is effective at reducing natural extrinsic stain over 12 weeks' twice daily brushing, in a population representative of the general public. The degree of stain removal was substantial: assuming the MLSI scale to be linear (which it is intended to be, although as a participative categorical scale it can only approximate to linearity^18^), over half the extrinsic tooth stain initially present was removed in the test group at 12 weeks, versus no significant stain removal in the reference group.

Interestingly, there was evidence of a stain removal effect of the reference group at 6 weeks. This may have been due to a ‘Hawthorne Effect’, such that participants brushed more diligently in the early weeks because they were participating in a clinical study.^21^ They appear to not have maintained this extra effort after 6 weeks as stain re‐accumulated to near prestudy levels by 12 weeks.

This study provides evidence that phytate acts like linear condensed polyphosphates in enhancing the stain removing ability of an otherwise conventional abrasive‐based dentifrice. It should be noted, however, that the abrasive systems of the two dentifrices involved in the study were not matched, so some of the enhanced stain removal of the test formulation would have originated from the higher abrasivity of the abrasive component in the test product. This was intended to reflect likely real‐world use of phytate: consumers desiring effective extrinsic tooth whitening are generally not also looking for a particularly low‐abrasivity toothpaste: a toothpaste in the ‘medium abrasivity’ RDA 70–150 range category^22^ is therefore appropriate. It is considered extremely unlikely that the degree of difference in stain removal measured between test and reference products seen in this study was due solely to the difference in the abrasive system.

Laboratory studies have shown that sodium phytate incorporated into a dentifrice formulation at the levels used in this study reduces stain accumulation and promotes stain removal (GSK Consumer Healthcare, data on file), as has been demonstrated for linear polyphosphates.^8^, ^9^ Hence, in this study, the inclusion of 0.85% w/w sodium phytate into a dentifrice formulation was expected to enhance the cleaning performance inherent to the toothpaste formulation by preventing new stain formation as well as removing existing stain. The twice‐daily brushing protocol allows both stain removal and stain prevention modes of action to contribute to measured stain levels.

There is also evidence that the chemical action of sodium phytate lends itself to extrinsic dental stain removal in areas where traditional toothbrushing is habitually missed (lingual surfaces) or areas that are hard to access (interproximal surfaces). These hard‐to‐reach areas demonstrated numerically the greatest extrinsic dental stain reductions. The ability to exert effects in hard‐to‐reach areas may prove beneficial regarding other dentifrice efficacy benefits. For example, it would be interesting to investigate whether the sodium phytate dentifrice developed for this study has anticalculus efficacy, following phytate's reported ability to reduce dental calculus formation in a mouthwash solution.^17^

Both products were generally well‐tolerated, indicated by the fact that there were very few AEs in either group, and there was no evidence that phytate at the level used in the test dentifrice had any adverse effects on oral soft tissues or teeth.

In conclusion, a statistically significant reduction in extrinsic dental stain was demonstrated between the experimental sodium phytate dentifrice compared to the reference control dentifrice, as indicated by both total and hard‐to‐reach area MLSI (*A* × *I*) scores, after 6 and 12 weeks of twice‐daily brushing.

## DISCLOSURE

This study was funded by GSK Consumer Healthcare. Kimberly Milleman and Jeffery Milleman are both Clinical Examiners and Directors at Salus Research, Indiana, USA, who have received funding from GSK Consumer Healthcare for this study. Jonathan Creeth and Gary Burnett are employees of GSK Consumer Healthcare and have no other conflicts of interest to report.
